# Strengthening the Structure of Public Health: Suggesting the Incorporation of ‘People’ Component as the ‘Seventh Block’

**DOI:** 10.5334/aogh.5012

**Published:** 2026-03-23

**Authors:** Delfin Lovelina Francis, Saravanan Sampoornam Pape Reddy

**Affiliations:** 1 Department of Public Health Dentistry, Saveetha Dental College and Hospitals, Saveetha University, SIMATS, Chennai, India; 2 Department of Periodontology, Army Dental Centre (Research and Referral), Delhi, India

**Keywords:** people-centred health systems, community engagement, systems thinking, health governance, WHO building blocks, health equity

## Abstract

*Background:* Health systems function as complex adaptive networks where institutions, professionals and citizens interact to maintain public health and social well-being. The World Health Organization conceptualised six foundational building blocks: service delivery, health workforce, information systems, access to essential medicines, financing and leadership/governance. While these components provide a valuable framework for strengthening systems, they insufficiently account for community dynamics and citizens’ roles in co-producing health. This article proposes the addition of a seventh building block, ‘People’, to formally recognize individuals, households and communities as active partners in generating and sustaining health outcomes.

*Methods:* A systems-thinking approach was used to analyse the interplay between institutional structures and community engagement, drawing on implementation theories and empirical evidence from India. The article explores how mechanisms under the National Health Mission, such as Village Health Sanitation and Nutrition Committees and Health and Wellness Centres, operationalize people-centred governance.

*Results:* Integrating ‘People’ as a distinct building block enhances accountability, equity and resilience by repositioning citizens as co-creators rather than passive beneficiaries. Empirical observations from India demonstrate how community-led governance and participatory platforms strengthen local health responses, particularly in vulnerable rural settings.

*Conclusion:* The proposed seventh building block, ‘People’, reframes health systems from institutional hierarchies into inclusive, democratic and adaptive networks. This reconceptualization is essential for addressing contemporary challenges, including pandemic recovery, climate-related health threats and the accelerating expansion of digital health.

## Introduction

The WHO’s Framework for Action 2007 articulated six building blocks to improve national health systems [1]. These foundational components were the analytic framework for diagnosing performance gaps, engineering interventions and measuring progress. Subsequently, the framework was accepted by governments, development partners and academicians around the world [2, 3]. The framework, nevertheless, has a strong unsaid bias towards institutional capacity and downplays the social mobilization and determinants of behaviour. Health does not just come from hospitals but from citizens, communities and policymakers every day [4–6].

The COVID-19 pandemic vividly illustrated the omissions. In India, community-driven networks (self-help groups, Accredited Social Health Activists [ASHAs] and volunteers) were instrumental in contact tracing, vaccination outreach among populations with lower vaccine literacy or vaccine hesitancy and risk communication. Where community participation was high, trust in public entities grew and service delivery was enhanced [7–9]. On the other hand, geographical localities with poor community connections were vulnerable to misinformation and vaccine hesitancy. The same trends were observed in other parts of the world, supporting the call for ‘people’-centred systems [10–13].

Systems thinking considers a health system as a complex adaptive network where feedback, learning and interdependencies determine performance. Learning becomes stagnant when the public is left out of feedback loops. The current review thus suggests extending the WHO’s six-block structure by including ‘People’ as a seventh block. This formula would ground the system in social concreteness, rebuild legitimacy and connect reforms to community priorities.

## Lacunae and Deficiencies in the Current Framework

Although mountain governance and investment provide an effective structure for managing land and capital, it is largely hierarchical. Its focus on the supply-side input elements, such as personnel, drugs and finance, places a managerial filter that can de-emphasize the power of users [14–16]. For example, the service delivery block focuses on institutional outputs (number of facilities and coverage rates) yet hardly mentions user experience, trust or empowerment. When speaking of a governance block, we usually refer to administrative control rather than citizen accountability.

According to the Indian government’s own National Health Policy (2017), community participation is key to accountability, but programme implementation continues to depend most on technocratic supervision rather than deliberative governance. Village Health, Sanitation and Nutrition Committees (VHSNCs), which are modelled under the National Health Mission (NHM), were intended to function as platforms for citizen oversight and local planning; however, their influence varies in states owing to limited financial autonomy and inadequate supportive capacity building [17, 18]. These dilemmas reflect the global epidemic of approaches that view communities as passive recipients, not as creators of health system change.

There is also an absence of focus on cultural competence. India is diverse linguistically, ethnically and religiously, so it needs a tailored service design and messaging strategies. Programmes designed without community participation often experience low utilization despite their technical soundness. The ‘People’ block would ensure contextual sensitivity and understand equity to be more than simply distributing resources evenly, but rather respecting differences [19–21].

Second, there is a complete absence of health literacy. It is not possible to engage meaningfully when the individual cannot understand and/or respond to health care information [22]. In India, research has shown adequate health literacy to be present in only one-third of the adult population for making necessary preventive choices, despite an alternative optimal ideal provision of health care [23, 24]. The deliberate omission of literacy from the WHO barrier list further illustrates this structural blind spot.

Third, accountability systems have largely been top-down constructs. In wealthier food cultures, data almost never come back down the system as feedback from the government to the community, and information is unbalanced. Trust evaporates among people who lack access to performance data and ways to seek redressal. The COVID-19 crisis has made palpable the power of transparency and citizen feedback as a way to rebuild confidence; some Indian states used real-time dashboards and held daily briefings (which answered publicly solicited questions), arguably operationalizing a two-way model that was faithful to the spirit of the ‘People’ block. The fact that these gaps remain indicates the need for more widespread recognition of system resilience. The addition of a ‘People’ block in the framework will address these ethical gaps.

## Inclusion of ‘People’ as the Seventh Block

**Figure 1:**
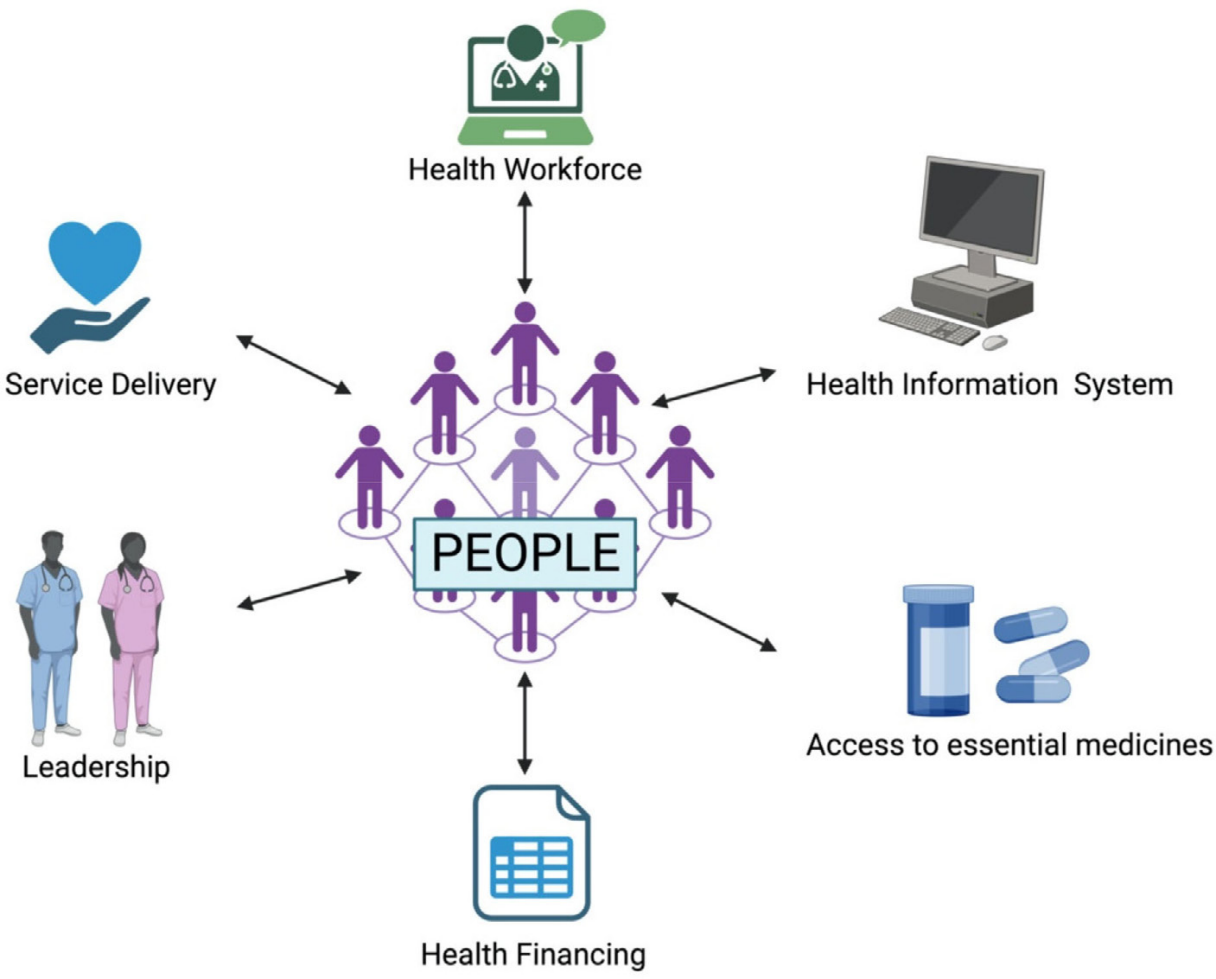
Incorporation of ‘People’ as the seventh block and its interdependency.

The ‘People’ component, as defined in the proposed model, acknowledges that populations are not passive targets of care but instead are active, ‘thinking’ subsystems within the system as a whole (Figure 1). It also makes conceptually explicit a feedback loop between institutional arrangements and populations, documenting the ways in which individuals, families and civil society organizations co-produce health. Within the Indian context, this concept is reflected in a number of existing programmes that already reflect people-centred ideals. In this context, the NHM created community-based mechanisms, such as Rogi Kalyan Samiti (patient-welfare committees) and VHSNCs, to decentralize decision-making and ensure that services delivered reflect local priorities. However, due to their under acknowledgement by the WHO framework, they rarely receive sufficient credit for evaluations or funding cycles. While many of these participatory mechanisms were initially designed for rural settings, urban community engagement structures have also emerged under the National Urban Health Mission. Urban health and nutrition days, mohalla clinics and urban community outreach programmes demonstrate how participatory governance models can also operate within densely populated metropolitan environments.

Integration of a formal ‘People’ block would redress this imbalance by embedding involvement. It would include health education and literacy efforts to foster citizen’s ability to make grounded choices [25]. In India, the School Health and Wellness Programme and community-based adolescent teachings component within Ayushman Bharat are some initial steps in that direction. These programmes foster an atmosphere conducive to informed citizenship by connecting schools, frontline workers and local health committees.

Cultural competency is also an important part of this block. The diversity of India dictates the need for a health system that caters to linguistic, regional and religious variety. For instance, the Indian state Kerala’s Tribal Health Mission and adaptations of the Janani Suraksha Yojana for indigenous persons in the Bihar state have shown how culturally sensitive design can increase trust and use [26–29]. The framework makes culture accountable, and culturalism moves from being an input innovation to a structural necessity in the ‘People’ tier. And with ‘People’ for transparency must come accountability in the flow of information. The Ayushman Bharat Digital Mission represents a model of data transparency in digital health governance. Through digital health IDs and personal health records, the system promotes data sovereignty by enabling citizens to control their health information while providing policymakers with real-time feedback on service quality and accessibility. These mechanisms reflect the People block’s orientation on co-responsibility with citizens as data providers and decision-makers as trustees of trust in society [30–34].

## Rationale of the ‘Seventh Block’

There are numerous reasons for there being seven, rather than six blocks on which the WHO model rests. A systems view of health care delivery systems is that they are complex adaptive systems, and the units themselves change as they interact. Without a human feedback loop, a system cannot learn and self-correct. Pooling together community experience using systematic means (participatory monitoring, digital platforms and citizen surveys) results in a distributed intelligence for the system, which makes it more robust to adapt.

From a governance viewpoint, involvement is not only consultative but constitutive of legitimacy. Contemporary theories of public administration stress co-production and, correspondingly, shared roles by the state and by citizens in their output [35–37]. India’s Health Management Information System (HMIS) now includes community sources from ASHA registers and village meetings more increasingly, signalling that bottom-up intelligence helps not only to target better but also in resource allocation.

From the perspective of equity and rights, participation is inherent to the right to health as articulated in constitutional jurisprudence and the Alma-Ata Declaration. Marginal groups, such as tribals, women and the urban poor, gain from a system that recognises their agency. Similarly, evaluations of community monitoring in Maharashtra, India, through the NHM, demonstrated a significant increase in maternal health indicators, complaint resolution and service utilization after villagers were trained to independently audit the facilities [38–40].

Internationally, the logic is similar. Ethiopia’s Health Extension Programme leverages local women’s associations for dissemination; Thailand’s National Health Assembly incorporates community perspectives into policymaking and Rwanda’s community-based insurance depends on group purchasing [41, 42]. These cases really demonstrate the argument that getting ‘people’ into some kind of formal institutional arrangement is going to create equity and efficiency.

## Role of Feedback Mechanisms

The channels of feedback build a link between the participation idea and practice. They are the sensors when systems watch, listen, understand and react to signals produced by society. In the absence of these loops, governance is not adaptable but reactive. India itself works on popular participatory monitoring under NHM and its implementation, as well as the CBMP (community-based monitoring and planning) process in states such as Maharashtra or Chhattisgarh, which have proved the effectiveness of structured feedback system to an extent. Villagers, health workers and local officials meet every three months to review service data that produce ‘report cards’ with criticism of shortfalls and suggestions for solutions. Assessments have demonstrated that CBMP responding sites are visited more by outpatients, acquire better drug availability and increase user satisfaction. Such incidents reflect the spirit of adding a new people’s block.

This evolution is thus being accelerated by digital tools, which enable a rapid feedback loop. For example, the portals, such as MyGov, eSanjeevani (National tele-consultation Service) and the grievance redressal portal of Ayushman Bharat, become the chip on which citizens are made to play by providing feedback and submitting grievances for redressal. When used as feedback, it is a structure of perpetual learning, and the Integrated Disease Surveillance Programme has now added social-media listening to help with early warnings of a future outbreak or other crisis, blurring even more than ever the lines between citizen and system.

Feedback mechanisms must operate in both directions. Communities require transparent access to performance information in order to build trust in health institutions. For example, the state of Kerala implemented publicly accessible COVID-19 dashboards that provided real-time data and addressed citizen questions during press briefings. Such practices demonstrate how transparent communication can strengthen accountability and public confidence. Evidence from low- and middle-income countries indicates that institutionalizing feedback systems improves health worker accountability and increases perceptions of fairness among citizens [43, 44].

## Capacity Building in Public Health Blocks

The building of capacity has to go beyond just the technical understanding to include populations and communities of people. Traditional frameworks have conceptualized ‘capacity’ as workforce training and infrastructure, but a people-focused perspective requires recognition of social capacity. In India, the NHM codified community participation with ASHAs, where one million women connect the households to the public health system. Their success shows that enabling local actors can change service delivery and accountability. Assessment of these workers has shown that ASHAs enhance immunization coverage, maternal health outcomes and household health literacy by transcending sociocultural barriers.

At the organizational level, community-based organizations and self-help groups have emerged as micro-institutions of governance. Self-help groups of women named as Deendayal Antyodaya Yojana—National Rural Livelihoods Mission—undertake health awareness programmes, nutritional supplementation and sanitation drives. These groups are an example of how capacity building in the ‘People’ block links livelihoods and health and strengthens the social determinants of health.

For the capacity development of programme systems, it is necessary to develop the practice of participatory monitoring. For instance, CBMP in India under the NHM provides a model where, through village health and sanitation, people compile health report cards that are used by governments for district planning meetings. The incorporation of such community findings into official review processes increases responsiveness and transparency [34, 35]. Similarly, the Ayushman Bharat Health and Health Wellness Centres (HWCs) institutionalize community outreach in the very architecture of primary care delivery. HWCs are knowledge nodes that can educate citizens about disease prevention, lifestyle modifications and health care entitlements, thereby building structural capacity that leads to resilience through informed participation.

This is rooted in the socio-ecological model, which acknowledges that health behaviour results from interactions at the personal, interpersonal, community and policy levels [36, 37]. Distributing individuals across all six existing blocks generates mutually reinforcing compacts, as engaged communities will raise the quality of service delivery, staff morale, information richness and equitable access to medicines while ensuring accountability. Trust, then, is the fulcrum of governance achieved by transparency and maintained through dialogue.

In the ‘People’ block, both at national and international levels, institutional training equals capacity building. A resilient system, promotes innovation and tolerates unevenness but leverages it to their advantage from the local knowledge [43–46]. This indicates that it is important to ensure that health worker training programmes impart not only participatory facilitation but also social mobilization and how to negotiate conflict and competencies that tend to receive less emphasis in education. By expanding capacity to include health systems learning from not just the data itself but discussing it, the ‘People’ block guarantees greater gains.

## Shifting Paradigms: From Provider-Centred to People-Centred Systems

The new architecture adds a ‘People’ block as a paradigm shift from provider-dominated bureaucracy to participatory governance. Human-centred care combines clinical efficacy with social justice, dignity and respectfulness. In India, this philosophy provides the foundation for the Ayushman Bharat reforms that combine financial protection with the revival of primary care. It recognizes that financial access always needs to go hand in hand with community trust by setting up HWCs and extending insurance.

People-centred systems redefine success metrics. Instead of counting the number of facilities or beds, they measure continuity of care, user satisfaction and empowerment. PM-JAY (Pradhan Mantri Jan Arogya Yojana) has systematized community awareness programmes, grievance redressal hotlines and beneficiary feedback portals to enforce responsiveness. These mechanisms echo international trends. Both Canada’s community health centres and Brazil’s Family Health Strategy show the better preventive results of decentralized, participatory models [47–49].

The experience of India in the context of the COVID-19 vaccination drive adequately supports this approach. Partnering with religious leaders, nongovernmental organizations and local influencers sent vaccine acceptance soaring. Misinformation was effectively counteracted by communication campaigns co-designed with communities. The ‘People’ block thus turns community relations from ad hoc outreach into institutional architecture and pandemic preparation for the next public health challenges.

## Policy Implications

To formally acknowledge the ‘People’ block would require not only a shift in policy but also an institutional overhaul. At the national level, India could incorporate community-engagement indicators into its HMIS and annual NHM programme reviews. Possible indicators could be the number of VHSNCs meeting, the percentage of women’s groups actively contributing to planning and the time taken to respond to citizens’ complaints. Dedicated budget lines for health literacy, supportive and participatory monitoring within the NHM and state plans need to be created. Making participation in fiscal policy enshrined means it is an investment in resilience rather than a discretionary cost [50].

The ‘People’ block can be consolidated by legislative instruments. The National Health Policy (2017) already requires ‘community participation in the achievement of health goals’, and an amendment or policy note could operationalize this through binding guidelines around representation in the district health societies. In states such as Kerala (India), which put local self-governments in control of primary health budgets, the process has helped deliver concrete gains for maternal and child health. To staff such models, all states will need the political will and capacity to scale up.

Lastly, the block of the masses supports good governance. By giving participation a formal shape, health systems recognize the right of citizens to have a voice and agency. This paradigm shifts accountability from oppressive surveillance into shared guardianship. If citizens see themselves as included, there is credible evidence that compliance with public health measures rises, corruption falls and policy legitimacy increases [51]. The moral epicentre of health-system governance, then, becomes the ‘People’ block.

At the global level, WHO and its partners could revise the Health Systems Assessment Approach to ensure these do not focus exclusively on secondary care-based measures of people-centeredness. Another component of the Global Health Observatory could be indices on empowerment, trust and inclusiveness. Organizations such as the Global Fund or GAVI may also stipulate evidence of the community’s governance processes in grant proposals. Such adaptation would be in line with the Framework on Integrated People- Centred Health Services (IPCHS 2023) and the Global Strategy for Health Systems Resilience (2022) [52–54].

For schools and colleges, this revolution entails curriculum changes. Civic engagement, participatory action research and systems thinking are part of medical education and public health curricula. If policy is to get off the ground, we need to train health workers to engage communities as partners, not recipients. The optimism stems partly from the current emphasis of the Indian Council of Medical Research on community-based implementation research.

## Bridging Global Agendas and Local Realities

The ‘People’ block would need to bridge planetary-scale institutional frameworks with on-the-ground experiences of everyday life if it is ever going to gain the traction necessary to steer in a different direction. And then actually making the ideas happen also means a genuine devolution of decisions and power, flexible funding for organizations with grants with prescriptive terms and respect for local knowledge systems. India’s highly centralized health administration, it must be noted, provides its own illustrations: Panchayats and VHSNCs, the local self-government entities that are ideally the locus of participation-led choices from below. But the question is how this can scale to a level where national priorities and local needs converge and whether such participation occurs not just for show but because information, financing and voice actually come within reach. This then locates a ‘People’ block within WHO’s range of action, the policy responsibilities for which run along a continuum from diplomacy to ward-level practice, and with community ownership, strengthening and sovereignty pivot. It captures the move from ‘doing for people’ to ‘working with people’, a reminder once again that sustainable public health rests on social legitimacy as it does on expert rigour.

## Conclusion

The WHO’s six-building-block blueprint has been the basis of health-systems-strengthening initiatives for more than a decade, but with new global realities (epidemics, climate emergencies, demographic shifts and digital disruption), it requires recalibration. The Indian experience demonstrates that community engagement mechanisms such as ASHAs, VHSNCs and HWCs can transform health systems into adaptive and participatory institutions. Integrating ‘People’ as a seventh building block does not replace the WHO framework but strengthens it by explicitly recognizing citizens as co-producers of health. Embedding community participation, health literacy and accountability within the system architecture will be essential for building resilient, equitable, and responsive health systems capable of addressing 21st-century global health challenges. Systems that co-produce with people are also more adaptive to uncertainty, keep reforms going and protect the universal right to health. It is not primarily a replacement for the WHO macro template, but rather a supplement to it. The suggested seventh block is ‘People’, recognizing that not only the way it interfaces but also the depth with which it penetrates its users are ways of measuring a health system. The embrace of that vision will help countries create health systems that are responsive, equitable, and high performing in the 21st century.

## Verification of all Authors

It is verified that all authors had access to the data and a role in writing the manuscript.
